# *Withania somnifera* (L.) Dunal (Ashwagandha); current understanding and future prospect as a potential drug candidate

**DOI:** 10.3389/fphar.2022.1029123

**Published:** 2022-12-12

**Authors:** Javeed Ahmad Bhat, Tahira Akther, Rauf Ahmad Najar, Faheem Rasool, Abid Hamid

**Affiliations:** ^1^ Cancer Pharmacology Division, CSIR-Indian Institute of Integrative Medicine, Jammu, India; ^2^ Department of Biochemistry and Biophysics, University of Rochester, Rochester, NY, United States; ^3^ B. S. Abdur Rahman Crescent Institute of Science and Technology, Chennai, India; ^4^ Department of Pediatrics (Neonatology), Lung Biology and Disease Program, University of Rochester Medical Center, Rochester, NY, United States; ^5^ Government College for Women, Jammu, Jammu and Kashmir, India; ^6^ Department of Biotechnology, School of Life Sciences, Central University of Kashmir, Srinagar, India

**Keywords:** cancer, neurodegenerative disorders, Alzheimer’s disease, *Withania somnifera*, stroke

## Abstract

Cancer and Neurodegenerative diseases are one of the most dreadful diseases to cure and chemotherapy has found a prime place in cancerous treatments while as different strategies have been tested in neurodegenerative diseases as well. However, due to adverse shortcomings like the resistance of cancerous cells and inefficiency in neurodegenerative disease, plant sources have always found a prime importance in medicinal use for decades, *Withania somnifera* (L.) Dunal (*W*. *somnifera*) is a well-known plant with medicinal use reported for centuries. It is commonly known as winter cherry or ashwagandha and is a prime source of pharmaceutically active compounds withanolides. In recent years research is being carried in understanding the extensive role of *W*. *somnifera* in cancer and neurological disorders. *W*. *somnifera* has been reported to be beneficial in DNA repair mechanisms; it is known for its cellular repairing properties and helps to prevent the apoptosis of normal cells. This review summarizes the potential properties and medicinal benefits of *W*. *somnifera* especially in cancer and neurodegenerative diseases. Available data suggest that *W*. *somnifera* is effective in controlling disease progressions and could be a potential therapeutic target benefiting human health status. The current review also discusses the traditional medicinal applications of *W*. *somnifera*, the experimental evidence supporting its therapeutical potential as well as obstacles that necessitate being overcome for *W*. *somnifera* to be evaluated as a curative agent in humans.

## Introduction

**FIGURE 1:**
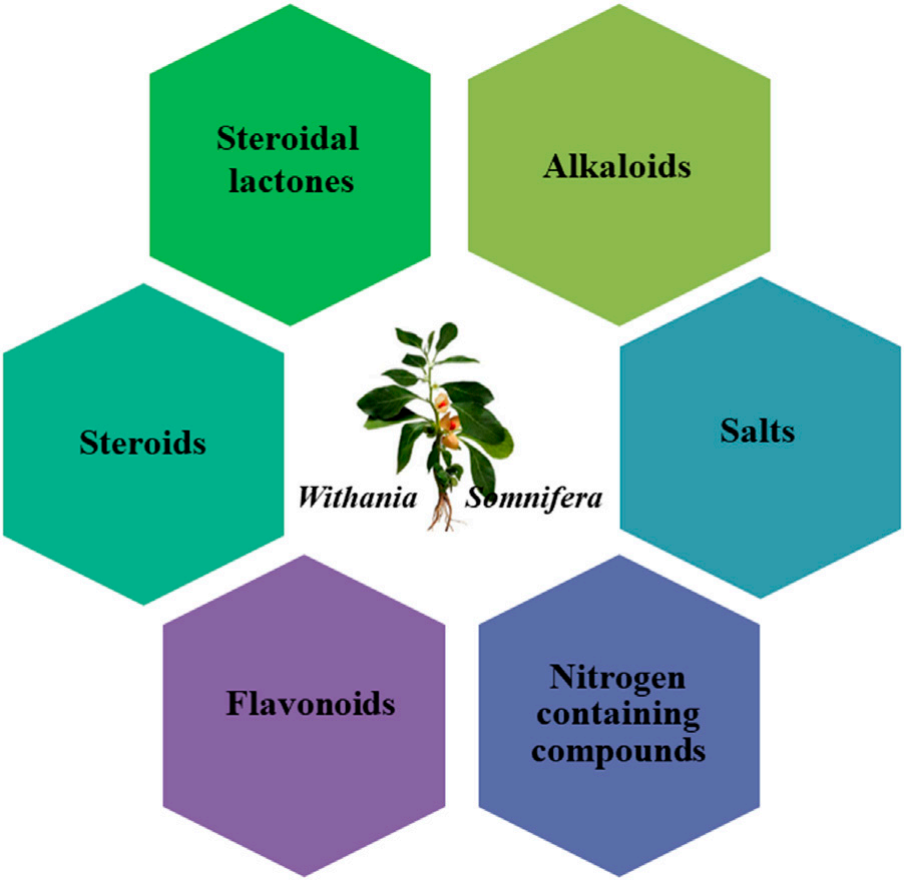
Phytochemicals from *Withania somnifera*.

**FIGURE 2:**
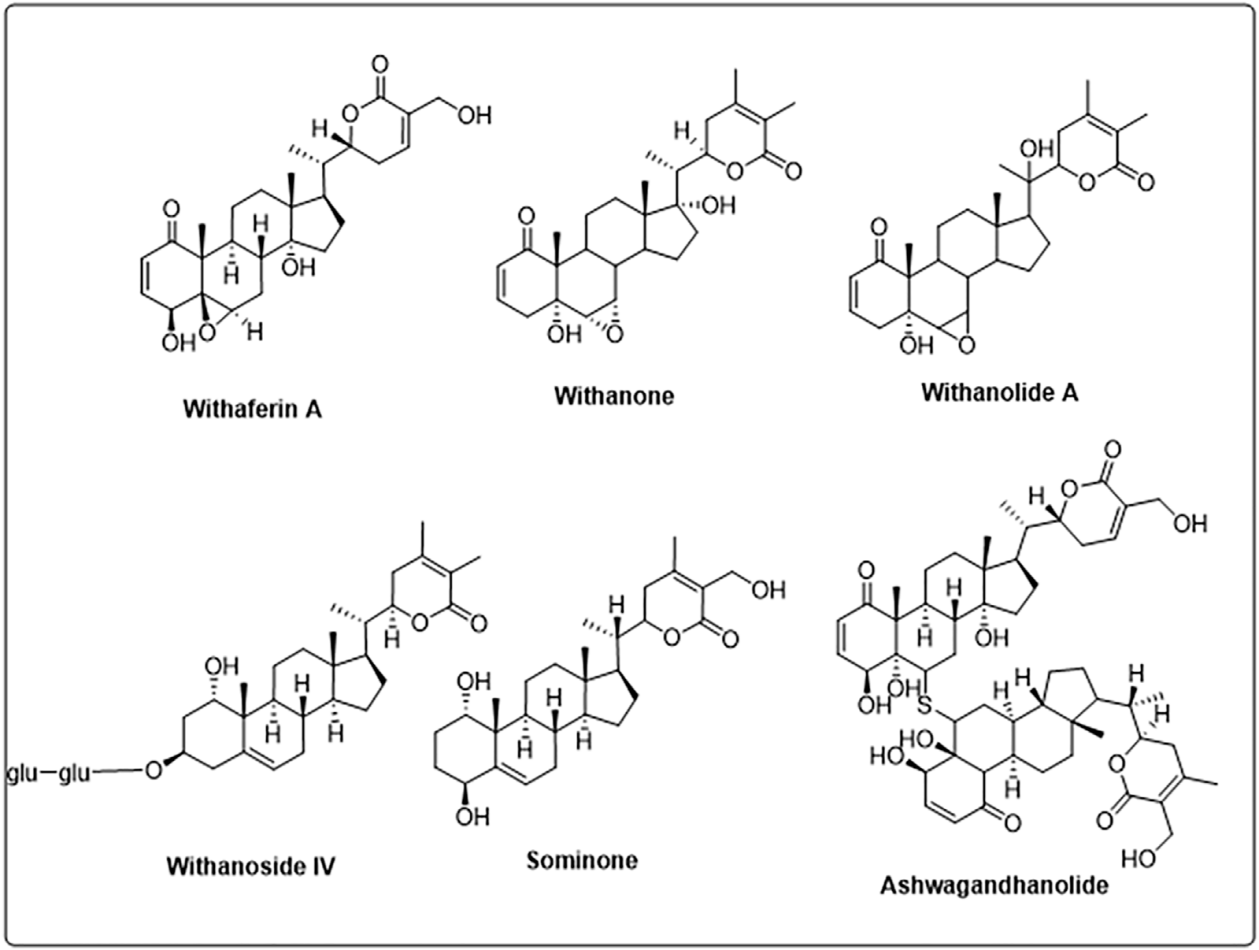
Structure of different phytochemicals from *Withania somnifera*.

A strong belief in traditional medicine has prompted the use of Nutraceuticals [like *Withania Somnifera* (L.) Dunal (*W*. *somnifera*), (Rivera et al., 2014; Heinrich et al., 2020), which has rigorously been used in Ayurveda] for enhancing general health status since ages and subsequent clinical evidence has turned this belief into practice, for instance at present dried powder capsules and alcohol derivatives are currently sold in the US market as drug supplement. This invite interest in exploring the efficacy of *W*. *somnifera* against challenging ailments including cancer chemoprevention which has tremendous room for discovering natural remedies even after a rich exploration in its direction for instance use of broccoli is suggested for the prevention of prostate cancer because of its bioactive constituent Sulforaphane. Ayurvedic drug Ashwagandha (*W*. *somnifera*), also known as Indian winter cherry and Indian ginseng, is a member of the Solanaceae family and has been used for thousands of years in India for its wide range of health benefits. Traditionally known as “ashwagandha,” was derived from the Sanskrit terms “ashva,” which means “horse,” and “gandha,” which means “smell,” and denotes the root’s aroma, which is similar to that of a horse. Sleep inducer is the species name “*somnifera,”* which highlights its critical role in stress relief. The plant is an upright, greyish, evergreen shrub with long tuberous roots, short stems, oblong, petiolate leaves, and greenish, bisexual flowers borne in the axils. Along with the drier regions of India, it can grow at high altitudes of 1700 m in the Himalayan region (mainly Himachal Pradesh, Uttarakhand, and Jammu and Kashmir). Despite the fact that there are 23 species of Withania identified, only *W*. *somnifera* and *Withania. Coagulans* (S) Dunal; *W. coagulans* (Rishyagandha) are thought to have therapeutic properties (Mirjalili et al., 2009b). Twenty-nine common metabolites generated from the leaf and root extracts have therapeutic significance in the form of flowers, roots, stems, and leaves. *W*. *somnifera* has been used as medicine since antiquity and has a long history, dating back to the year 6,000. Withanolides (Withaferin A, Withanolide A, Withanone), sitoindosides, Withanosides, and other alkaloids are the plant’s main metabolites, and they may have therapeutic and medical benefits (Figures 1, 2). It is used to treat a wide range of clinical disorders such as reducing blood sugar levels, depression, and stress, increasing energy, and enhancing cognitive function. In addition to its palliative effects, such as analgesic, rejuvenating, regenerating, and growth-promoting properties, this medicinal plant has been reported to be effective against epilepsy, inflammation, arthritis, depression, coagulation issues, free radicals, diabetes, and pyrexia. These therapeutic effects are partly attributable to *W*. *somnifera’s* potential to decrease reactive oxygen species, alter mitochondrial activity, regulate apoptosis, lower inflammation, and improve endothelial function by elevating the defense system. The first published report on the antibacterial activity of the *W*. *somnifera* plant dates back to 1958 (KURUP, 1958); later on, in 1960, the effect of whole plant extract of *W*. *somnifera* on the central nervous system (CNS) and skeletal muscles (Malhotra et al., 1960). Dhalla et al. (1961) first reported the chemotherapeutic properties of the extracts isolated from the leaves of *W*. *somnifera*. In a transplantable mouse tumor called Sarcoma 180, Devi et al., 1992 revealed for the first time in 1992 the *in vivo* growth inhibitory effects of plant root extracts of *W*. *somnifera’s*.

## *Withania somnifera* in ayurveda

Although *W*. *somnifera* is more frequently employed in conventional medicine, *W. coagulans* is also used in some particular preparations (Upadhyay and Gupta, 2011). In more than 200 Ayurvedic compositions, *W*. *somnifera* roots are used. The powdered root of the *W*. *somnifera* plant known as ashwagandha is frequently used to cure several diseases. It is additionally combined with other compounds. The main ingredient in Saraswati churna, an herbal powder mixture intended to treat neurological problems, is *W*. *somnifera*. Another product that contains *W*. *somnifera* is ashwagandhadhi lehyam, which is generally used as an energy booster, a cure for male impotence, and a supplement for general rejuvenation (Rasheed et al., 2013). Even while these applications may appear to be very different, it’s conceivable that certain ratios and combinations with the other substances could provide very different results. It’s interesting to note that traditional medical treatments only use the plant’s root. Researchers in alternative medicine have lately examined the use of *W*. *somnifera* in Ayurvedic formulations and found that standardization, phytochemical screens, and testing for pathogen/heavy metal contamination can considerably enhance the effects of Ashwagandadhi lehyam. *W*. *somnifera* containing ayurvedic preparations are used as analgesics for a variety of musculoskeletal disorders (including arthritis and rheumatism), some types of hypertension, for inducing sex and boosting sperm counts, in gynecological practice for vaginitis, and during pregnancy for the development of the breasts (Mishra et al., 2000).

## *Withania somnifera* and cancer

Cancer is a term used to refer to a wide range of illnesses and is distinguished by the rapid development of aberrant cells that proliferate uncontrollably. These aberrant cells typically upset the balance between cell proliferation and cell death, resulting in benign tumors that subsequently develop invasive characteristics and manifest symptoms of the disease that range from benign to metastatic (Bhat et al., 2017; Bisoyi, 2022). Most metastatic-stage malignancies are incurable and are the main reason for cancer-related deaths (Bano et al., 2020; Qadir Bhat et al., 2022). Genetic and environmental factors play a major role in how a normal cell becomes malignant. Physical carcinogens like UV and ionizing radiation, chemical carcinogens like asbestos, aflatoxin, and arsenic, and biological carcinogens including parasitic, bacterial, and viral infections are examples of external agents (Parsa, 2012). Mutations in the genes primarily responsible for cell survival, proliferation, and growth are brought on by these stimuli. One in six fatalities in 2018 were attributed to cancer, according to the WHO’s IARC (International Agency for Research on Cancer), which translates to 9.6 and 10 million deaths in 2018 and 2020, respectively. According to the IARC report 2020, there will be 30.2 million new cases of cancer worldwide by the year 2040, up from 19.3 million in 2020. India was expected to have 1,392,179 cancer patients by the year 2020, with men making up 679,421 (94.1 per 100,000) and women making up 712,758. (103.6 per 100,000). Lung, oral, prostate, tongue, and stomach cancers, which account for around 36% of all cancer cases in men, are the most often diagnosed cancers in these individuals, while as breast, cervix, uteri and lung constitute about 53% of all cancers in females (Mathur et al., 2020; Sung et al., 2021).

Of the many voids that still demand to be attended in the direction of research for battling and combating cancer is the safety and accuracy of drugs, a treatment that is free of side effects, being more precise. Heterogeneity exhibited by cancer cells, and a complexity associated with tumor interactions stands as a roadblock in the direction of efficacy of various therapies and this effort for exploring such drugs, has unraveled many naturally existing materials with anti-cancer activity and extracts of *W*. *somnifera* is a fine example.

*W*. *somnifera* is well studied herb and mounting evidence from *in-vitro* and *in-vivo* studies suggest that *W*. *somnifera* hosts anti-tumorigenic properties. The first experimental evidence in 1967, showed that *W*. *somnifera* root extract lowered cancer incidence *in-vivo* (Shohat et al., 1967) which centered research focus and interest in *W*. *somnifera* to explore its anti-tumor activity and direct towards its therapeutic potential till date. The anti-tumorigenic potential of *W*. *somnifera* prevail to its activity to speed up apoptotic cascade in cancer cells. The potential of cancer chemoprevention, cell survival and the activation of pro-apoptotic pathways needs successful reversal of the carcinogenesis that requires the early clearance or destruction of impaired cells. A plethora of *in vitro* evidence exists that validated that *W*. *somnifera* and its constituents like withanolides induce apoptosis (Srinivasan et al., 2007; Malik et al., 2009; Das et al., 2014). The earliest study of *W*. *somnifera* assess the potential role of leaf extract to inhibit tumor formation in mice which was subcutaneously injected with fibrosarcoma HT1080 cells (Widodo et al, 2007). In this study, it was concluded that mice treated with leaf extract (0.3 ml of 24 μg/ml extract in cell growth medium) attenuated tumor size *via* upregulation of p53. In another *in vivo* study it was observed that introduction of *W*. *somnifera* by intraperitoneal route (4 mg/kg body weight) 5 times for 2 weeks markedly suppresses MDA-MB-231 tumor weight as well as exhibited reduced cell proliferation and increased apoptosis compared with tumors from control mice (Stan et al., 2008b)In cervical cancer using xenograft mouse model, it was observed that i,p treatment of withaferin A (8 mg/kg body weight) for 6 weeks resulted in 70% reduction in tumor size compared to controls as well as heightened expression of p53 and lowered expression of pro-caspase 3/Bcl2(Munagala et al., 2011). In a study conducted on a xenograft mouse model of lung cancer, i,p administration of withaferin A (4 mg/kg body weight) reduced tumor volume by 60% (Gupta et al., 2012). The oral administration of withaferin A (5 mg/kg) prevents prostate adenocarcinoma, inhibit AKT signaling, and activate Foxo3a-Par-4-induced cell death and EMT markers (vimentin, β-catenin, and snail and upregulate E-cadherin) (Suman et al., 2016).

A wide range of cancers are treated using various therapeutics, either alone or in combination (Table 1). The potential of effective tumor treatments to interfere with tumor replication process, return normal cells to homeostasis with a minimum of side effects, and exhibit selectivity are among their most desirable pharmacological properties (Hussain et al., 2019; Nalli et al., 2019). The heterogeneity of cancer cells and complicated tumor interactions, which lead to clonal selection of a drug-resistant cell population, continue to limit the efficacy of many therapies despite significant efforts and the introduction of numerous chemotherapeutic drugs in the clinic. Due to their ability to target several cancer hallmarks, such as cell proliferation and death resistance, replicative capacity, and apoptosis, natural pharmaceutical compounds have attracted interest as chemotherapeutic agents to prevent or overcome treatment resistance.

**TABLE 1:** Preclinical status of Withaferin A in cancer.

Source	Cancer	Potential mechanism	References
Withaferin A	Lung cancer	Cell cycle arrest; decreases PI3K/Akt pathway	Cai et al. (2014)
Withaferin A	Lung cancer	Decreased TGF-and TNF- induced EMT; decreased nuclear translocation of Smad 2/3 and NF-κB	Kyakulaga et al. (2018)
Withaferin A	Lung cancer	Increased ROS, autophagy, and apoptosis; decreased mTOR/STAT3 signaling	Hsu et al. (2019)
Withaferin A	Leukemia	Increased Apoptosis; increased G2/M phase cell cycle arrest and increased ROS	Okamoto et al. (2016)
Withaferin A	Glioblastomas	Decreased Cell proliferation; increased G2/M phase cell cycle arrest; increased ROS generation; decreased Akt/mTOR and MAPK pathway	Grogan et al. (2013)
Withaferin A	Breast cancer	Decreased mammosphere formation, decreased ALDH1 activity and bCSCs	Kim and Singh (2014)
Withaferin A	Breast cancer	Decreased Cell migration, EMT and invasion; decreased IL6 induced STAT3 activation; increased Notch2 and Notch4 and decreased mitochondrial membrane potential	(Widodo et al. 2007; Lee et al. 2010)
Withaferin A	Breast cancer	Increased G2/M phase cell cycle arrest and ROS generation and apoptosis, decreased ER-a, XIAP, cIAP-2 and survivin	Lee et al. (2010)
Withaferin A	Neuroblastomas	Decreased cell proliferation; increased G0/G1 cell cycle arrest; decreased Cyclin D1 and p-Akt, PSA-NCAM, Bcl-xL, MMP-2, MMP-9	(Chang et al. 2016; Kataria et al. 2016)
Withaferin A	Prostate	Decreased cell proliferation; increased G2/M Phase cell cycle arrest and ROS and autophagy	Nishikawa et al. (2015)
Withaferin A	Ovarian cancer	Decreased cell proliferation; increased apoptosis; ROS and G2/M cell cycle arrest; decreased Notch1, Notch2, otch3, Bcl-2, Akt	Fong et al. (2012)
Withaferin A	Gastric cancer	Decreased cell viability; increased Apoptosis; G2/M cell cycle arrest and ROS; decreased Cell migration and invasion	Kim et al. (2017)
Withaferin A	HFD-induced obese mice and Human Umbilical vein endothelial cells (HUVECs), Mouse, murine fibrosarcoma	Found to be anti-obesity *via* reduction in COX2, NF-kB, TNF-α, inflammation, insulin resistance and oxidative stress. It was also found to be anti-inflammatory in later models *via* the downregulation of C- JNK, ERK-1/2, P38, IL-1_β_ like proteins	Heyninck et al. (2014); Abu Bakar et al. (2019)
Withaferin A	Human Melanoma cells (M14, Lu1205, SK28) and Breast cancer cell lines (MDA-Mb231 and MCf-7)	Found to be anti-cancerous *via* upregulation of apoptosis (ROS induced) by decreasing the Bax/Bcl2 and Bcl2/Bim ratio. However, in breast cancer upregulation of caspase-9 and 3 along with PARP was found to be the vital components contributing to anticancer nature of WA.	(Stan et al. 2008a; Mayola et al. 2011)
Withaferin A	Xenograft (Breast cancer) and transgenic mice models	Found to be anti-cancerous *via* upregulation of ERK/RSK axis, DR-5 (death receptor 5), ETS domain containing protein-1, and CAT/CHOP proteins	Nagalingam et al. (2014)
Withaferin A	Human Laryngeal Carcinoma (Hep 2 cell line) and Renal cancer (Caki cell line)	Found to be anti-cancerous *via* downregulation of cell cycle arrest with possible blockage of angiogenesis and downregulation of STAT-3 pathway and upregulation of GRP-78 and CHOP proteins are thought to be main player in Caki cells	Mathur et al. (2006); Choi et al. (2011)

Results from various animal studies and cell culture have highlighted anti-tumorigenic properties of *W*. *somnifera*. First demonstration of anti-cancerous properties of *W. somnifera* dates back to 1967 when root extracts were reported to lower incidence of cancer *in vivo*. This has attracted a trail of research interests and a timely revelation of its anti-tumorigenic properties. A hint of it can be inferred from a seemingly increase in publication number citing extracts of the plant over the past decade. It has been unraveled that anti-cancerous activity is not restricted to just roots but extends to scarcely used parts of the plant like leaf extracts. In a study by Wadhwa et al. (2013) a considerable anticancer activity was seen in water extracts of leaves of the plant. Metastasis, which is a hallmark of some cancer cells stands as a big hurdle in cancer therapies, and Withania is a good substitute to such therapies to curb the spread of cancer cells. Using vimentin as a pro metastatic protein, a decreased cell motility of breast tumor has been witnessed using different formulations of *W*. *somnifera*. Researchers went ahead and proved that inhibition of metastasis of breast cancer is associated with administration of root extracts of the plant with least severe effects in rats. A clear suggestion about potential of *W*. *somnifera* in regulation of G2/M cell cycle of tumor cells came out with an observation against prostate cancer that use of *W*. *somnifera* leads to metabolic inactivation of Cdc2 catastrophe and a subsequent cell death. Alkaloid extracts of *W*. *somnifera* mediates disruption of mitotic procedure by binding at the site of BIR5 protein and hence displays a potential for antitumor activity.

Although there are several chemoprotective medications available for the treatment of cancer, the most of them are quite expensive and have numerous adverse effects (Bayat Mokhtari et al., 2017). Therefore, it is vitally necessary to look for promising natural and cost-effective medications with minimal side effects to lower the morbidity rate. Cancer treatment with plant-based immunoprotective drugs is seen to be the most practical option (Ahmad et al., 2020). For these goals, a variety of plants with dietary origins, such as cruciferous vegetables, are employed (Greenwell and Rahman, 2015). They are more durable and have very few side effects. As a result, there is an urgent need for natural medicines that can stop the cancer-causing process.

The use of *W*. *somnifera* in cancer therapy has been extensively studied during the past 20 years. Initially, it was mostly used to treat concerns with conception and reproductive healthcare, but now, it is also used to prevent ageing, calm anxiety, increase vital fluid, semen, cell, blood, and lymph production, treat other health issues, and nourish various body parts (Singh et al., 2021).

The extensive phytochemistry of *W*. *somnifera* is what accounts for its multimodal actions. The therapeutic benefits of several metabolites are being researched. Withanolides are the main metabolites that demonstrate a variety of actions. The triterpenoids that make up the withanolides steroidal lactone backbone have about 28 carbons (Saggam et al., 2020). Phytochemicals known as withanolides are derived from the phytochemical ergostane, which is synthesized by plants utilizing isoprene units as precursor (Saleem et al., 2020). Additionally, sitoindosides is the name given to the glycolated withanolides. Carbon number 27 of Withaferin A has a β-D-glucopyranosyl residue that comes from sitoindoside IX (27-O-glucosylwithaferin A) (Mirjalili et al., 2009a). Sitoindoside X is created chemically by attaching palmitic acid to 27-O-glucosylwithaferin A. Similar to this, triethylene glycol and its derivatives have recently been discovered to be active *W. somnifera* components.

**FIGURE 3:**
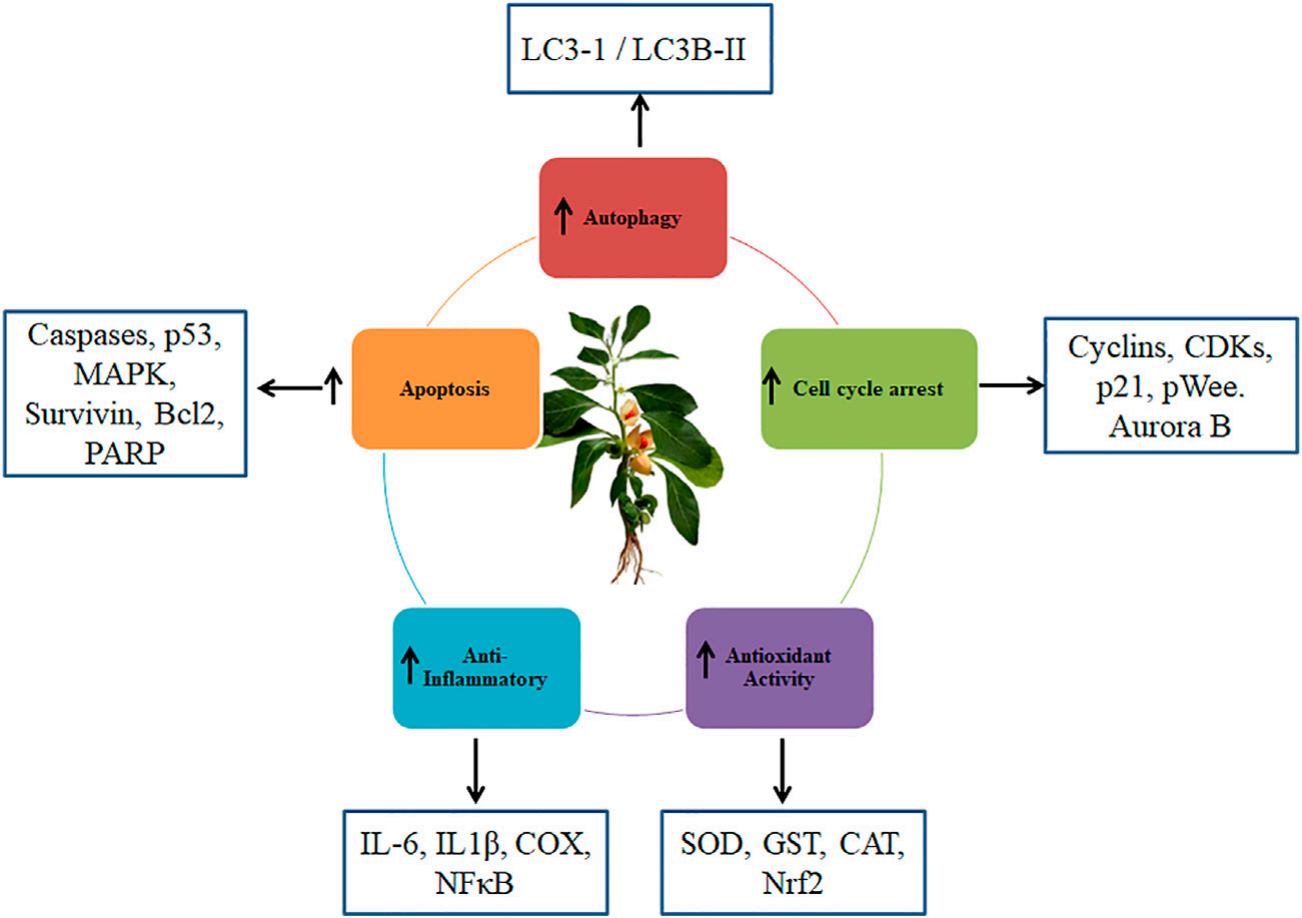
Effect of *Withania somnifera* on different pathways involved in Cancer.

Numerous studies have reported that withaferin-A exerts anti-tumor activity through a number of mechanisms, including activation of the tumor suppressor protein (p53), antioxidant (ROS) signalling, activation of apoptosis, inhibition of epithelial-mesenchymal transition (EMT) signaling, decrease in phosphoinositide 3-kinase (PI3K) signaling, decrease in angiogenesis inhibition of Nuclear factor kappa B (NF-Kappa β) activation, decrease in notch signalling, altered cytoskeletal architecture, and downregulation of cell cycle proteins (cyclin B1, cyclin A, cdk2, expression of p-cdc), of oncoproteins, activation of tumor suppressor and anti-apoptotic proteins such as p53, Bcl 2, BAX, caspase 3, and cleaved poly-(ADP-ribose)-polymerase PARP (Sharma et al., 2014; Ahmad et al., 2017; Liu et al., 2019; Mallipeddi et al., 2021; Surya et al., 2021). *W*. *somnifera* extracts have shown potential for the treatment of a wide range of cancers, including those of the skin, breast, colon, liver and pancreatic (Najar et al., 2018) (Figure 3). *W*. *somnifera* root extract “priming” in HT-29 colon cancer cells boosted the effectiveness of the chemotherapy drug cisplatin which leads to mitochondrial dysfunction *via* increased ROS production (Henley et al., 2017). Leaf extracts of *W*. *somnifera* selectively kill the tumor cells by activating tumor suppressor protein p53 (Widodo et al., 2007). In pancreatic cancer cells, *W*. *somnifera* and some of its withanolides are reported to bind to HSP90 and decrease its chaperone function *via* an ATP-dependent mechanism (Yu et al., 2010b). The apoptosis induction, which is characterized by DNA condensation, cytoplasmic histone-associated DNA fragmentation, and cleavage of PARP, was connected with the withaferin A -mediated suppression of breast cancer cell survival (Dar et al., 2015, 2017; Ahmad and Dar, 2017). Withaferin A therapy decreases the expression of the NF-B and mTOR pathways in MDA-MB-231 and MCF-7 human breast cancer cell lines in a dose-dependent manner. These modifications significantly induced apoptosis, which was found to be correlated with upregulated Bax, Bim-s, Bim-L, Bim-EL and downregulated Bcl-2 protein expression. *In vivo* MDA-MB-231 xenograft suppression by withaferin A is accompanied by decreased cellular proliferation and enhanced apoptosis by FOXO3a and Bim (Stan et al., 2008b). Withaferin A shows anticancer activity in pancreatic cancer cell lines and *in vivo* pancreatic cancer xenografts *via* inhibition of Hsp90 in an ATP-independent manner which induces protein degradation and disrupts the Hsp90-Cdc37 interaction (Yu et al., 2010a). Combination of Withaferin A and oxaliplatin were used together to treat pancreatic cancer (PanCa), this led to intracellular ROS accumulation, which was correlated with Akt downregulation and apoptotic cell death (Bhat et al., 2021). This offered the strongest proof to yet of the combination therapy of withaferin A and oxaliplatin’s anticancer efficacy in PanCa therapy (Li et al., 2015). Withaferin A inhibits the progression of pancreatitis by blocking Endoplasmic Reticulum (ER) stress and the NLRP3 inflammasome (Kanak et al., 2017). Water extracts of *W*. *somnifera* has anticancer and antioxidant properties against Hepatocellular carcinoma (HCC) cell line HepG2 (Lin et al., 2017).

## Role of *Withania somnifera* in various neurodegenerative disorders

*W. somnifera* has been thoroughly studied during past few decades especially in neurodegenerative diseases (Table 2). Let’s analyze *W*. *somnifera* in neurodegenerative diseases in detail:

**TABLE 2:** Preclinical status of *Withania Sominifera* in neurodegenerative diseases.

Source	Model tested	Potential mechanism	References
Withania Somnifera plant extract	Male Wistar Rats	Showed anti-Alzheimer’s activity by downregulating acetyl cholinesterase	Das et al. (2014)
Withania Somnifera plant extract	Amyloid-_β_ marker thioflavin-T	Anti-amyloidogenic *via* reduction in amyloid beta	Das et al. (2021)
Root extract of Withania Somnifera	SH-SY5Y cell line	Found to be anti-amyloidogenic *via* reduction in Aβ40	Tiwari et al. (2018)
Root extract of Withania Somnifera	CHME5 microglial cell line	Found to be anti-inflammatory *via* the downregulation of JUN, NF-kB, and STAT gene apart from the downregulation of IL-1β as well	Atluri et al. (2020)
Aqueous root extract of Withania Somnifera	Rat pheochromocytoma (PC12) cell line	Showed anti-Alzheimer’s activity by downregulating H202- and Aβ induced toxicity	Kumar et al. (2010b)
Aqueous methanol extract of Withania Somnifera roots	Mice	Reversed anti-AChE activity *via* enhancing Ach, choline acetyltransferase and ChAT activity in globus pallidus and lateral septum	Uddin et al. (2019)

## Alzheimer’s disease

The most prevalent cause of dementia in senior people is Alzheimer’s disease (AD), a neurodegenerative condition that progresses over time and is histochemically characterized by extracellular amyloid beta (Aβ) protein deposits and intracellular neurofibrillary tangles in the cortical and limbic regions (Serrano-Pozo et al., 2011). Experimental models of AD’s behavioral impairments and clinical signs have been demonstrated to be reversed by *W*. *somnifera* extract. *W*. *somnifera* mediated reversal of amyloid induced toxicity in SK-N-MC neuronal cells and also reversed amyloid-induced reduction in spine density, spine area, spine length, and spine number, indicating protective impact of *W*. *somnifera* in AD (Dar and Ahmad, 2020). Moreover, *W*. *somnifera* supplementation reverses the effect of amyloid treatment in neuronal cells by inhibiting the acetylcholinesterase activity and reduction of amyloid-β internalization (Sehgal et al., 2012; Kurapati et al., 2013). Bioinformatics studies have revealed the mechanism by which inhibitory effect of Withanolide A in AD. Withanolide A binds to the different residues of acetylcholinesterase enzyme (such as Thr78, Trp81, Ser120, and His442). These residues are present in the active site and play a pivotal role in proper functioning of acetylcholinesterase enzyme (Grover et al., 2012). Cell death caused by the amyloid toxicity in PC-12 cells is abrogated by the treatment of Withaferin A and C. These Withanamides blocks the active site of β-amyloid and inhibits the fibril formation (Jayaprakasam et al., 2010). Root extracts of *W. somnifera* orally administered to Alzheimer’s transgenic mice reverse the accumulation of Aβ and behavioral deficits by upregulation of low density lipoprotein receptor-related protein (LRP) (Sehgal et al., 2012). Behavioral deficits or cognitive defects induced by Bisphenol A (BPA) and ibotenic in Swiss albino mice are reversed by the treatment of *W*. *somnifera* by restoring NMDA receptors (Singh et al., 2011; Birla et al., 2019). *W. somnifera’s* root aqueous extract functions as a neuroprotective agent by shielding PC-12 cells from the cytotoxicity brought on by Aβ (1–42) and H_2_O_2_ (Kumar et al., 2010a). Withanolide A also protects the AD by increasing the expression of neuroprotective protein hemeoxygenase-1 (Nitti et al., 2018). Withanolide A may benefit AD by promoting neuritogenic activity and inhibiting secretase activity (Strooper et al., 2010). People with MCI may benefit from Ashwagandha’s potential to improve executive function, attention, and the speed at which information is processed (Choudhary et al., 2017). *W. somnifera* extracts can enhance memory and cognitive performance by modifying cholinergic neurotransmission (Pingali et al., 2014). In addition, high resolution Q-TOF/MS research has demonstrated that the Withanamides in *W*. *somnifera* fruit extract crossed the blood-brain barrier in mice after intraperitoneal administration, suggesting that oral administration of the extract could result in effects similar to those seen after intraperitoneal administration because the extract has functionalities that are both lipophilic and hydrophilic and easily crosses membranes (Vareed et al., 2014).

## Parkinson’s disease

Parkinson’s disease (PD) is a neurodegenerative motor disorder marked by the loss of dopaminergic neurons in the substantia nigra (Wongtrakul et al., 2021). Development of PD is caused by decreased dopamine levels in brain areas controlling motor activities (Mazzoni et al., 2012). PD has been linked to impaired anti-oxidative defense mechanisms and increased production of oxidative free radicals. Dysregulation of glutathione peroxidase (GPX), catalase, and superoxide dismutase (SOD) increases harmful free radicals build up and the disease progresses in a degenerative way (Maxwell, 1995). Pretreatment of *W*. *somnifera* extracts in hydroxyl dopamine (6-OHDA) Parkinson disease rat model does not alter the expression of antioxidant enzymes such as catalase, tyrosine hydroxylase, glutathione peroxidase, and SOD (Ahmad et al., 2005). The 1-methyl-4-phenyl-1,2,3,6-tetrahydropyridine (MPTP)PD model treated with *W*. *somnifera* (100 mg/kg body weight) for 7 or 28 days showed higher expression of DA, HVA, and DOPAC when compared with control MPTP PD model (RajaSankar et al., 2009). Ethanolic root extracts of *W*. *somnifera* treatment provides nigrostriatal dopaminergic neuroprotection against MB–PQ induced Parkinsonism by the reducing the expression of iNOS (an oxidative stress marker oxidative stress) and antiapoptotic proteins such as Bax and Bcl-2 (Prakash et al., 2014).

## Huntington’s disease

Huntington’s disease (HD) is a deadly neurodegenerative disorder that results from the destruction of neurons in the basal ganglia. HD selectively targets striatal spiny projection neurons (Choudhary et al., 2013). Progressive motor dysfunction, such as chorea and dystonia, emotional issues, memory problems, and weight loss are the hallmarks of HD. It is generally known that GABAergic system has a role in the pathogenesis of HD, and it is also well known that *W*. *somnifera* acts through GABAergic system. In mice treated with 3-nitropropionic acid (3-NP), *W*. *somnifera* root extract pretreatment dramatically recovered glutathione enzyme level system, acetyl cholinesterase enzyme activity, and cognitive function (Kumar and Kumar, 2009). In a 3-NP-induced model of HD, *W*. *somnifera* root extract has been shown to significantly improve cognitive behavior (as measured by the Morris Water Maze and Elevated Plus Maze tests) and motor (impairment of muscle activity as measured by the Rotarod and Limb Withdrawal Tests) activities. This improvement has been attributed to the inhibition of oxidative stress, restoration of antioxidant status, and enhancement of acetylcholinesterase enzyme activity on *W*. *somnifera* supplementation (Paul et al., 2021).

## Cerebral ischemia

Stroke is one of the leading causes of brain injury for millions of individuals worldwide. About 87% of strokes are ischemic strokes (Go et al., 2014). *W*. *somnifera* pre-supplementation (50 mg/kg) reduced the reperfusion injury-induced biochemical and histological changes in a rat model of bilateral common carotid artery blockage. In a different study, it was discovered that pre-supplementing with *W*. *somnifera* reduced oxidative stress, lesion volume, and restored neurological impairments in the middle cerebral artery occlusion (MCAO) stroke model (Choudhary et al., 2013). Additionally, *W*. *somnifera* dramatically reduced the size of the cerebral infarct and improved the histological changes in MCAO mice. Hemeoxygenase 1 (HO1) expression was found to be upregulated by *W*. *somnifera* treatment, which also attenuated the expression of the PARP1 *via* the PARP1-AIF pathway, preventing the nuclear translocation of apoptosis-inducing factor (AIF), suggesting involvement of anti-apoptotic pathways and angiogenesis (Raghavan and Shah, 2015). Stroke, multiple sclerosis, brain injuries, and neurodegenerative diseases have all been linked to glutamate neurotoxicity. Inhibition of glutamate-induced neurotoxicity by a water extract from the leaves of *W*. *somnifera* has been seen in retinoic acid differentiated rat glioma (C6) and human neuroblastoma (IMR-32) cells (Kataria et al., 2012). Withanolide A reverses the hippocampus’s hypoxia-induced glutathione depletion and slows the progression of neurodegeneration. Additionally, withanolide A promoted glutamate-cysteine ligase (GCLC) levels through the Nrf2 pathway in a corticosteroid-dependent manner, increasing glutathione production in neuronal cells (Baitharu et al., 2014).

## Epilepsy

**FIGURE 4:**
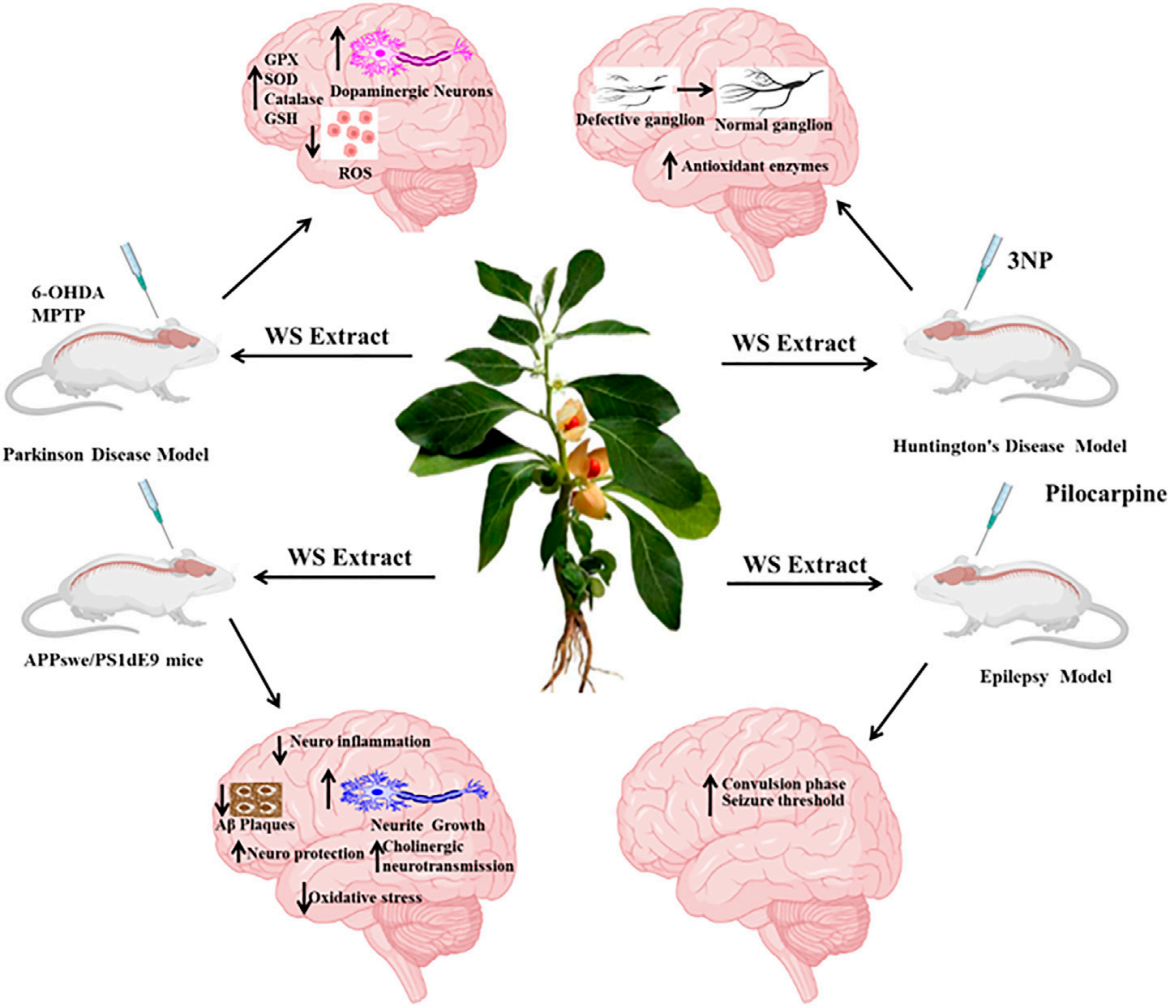
Effect of *Withania somnifera* on different neurodegenerative diseases.

**FIGURE 5:**
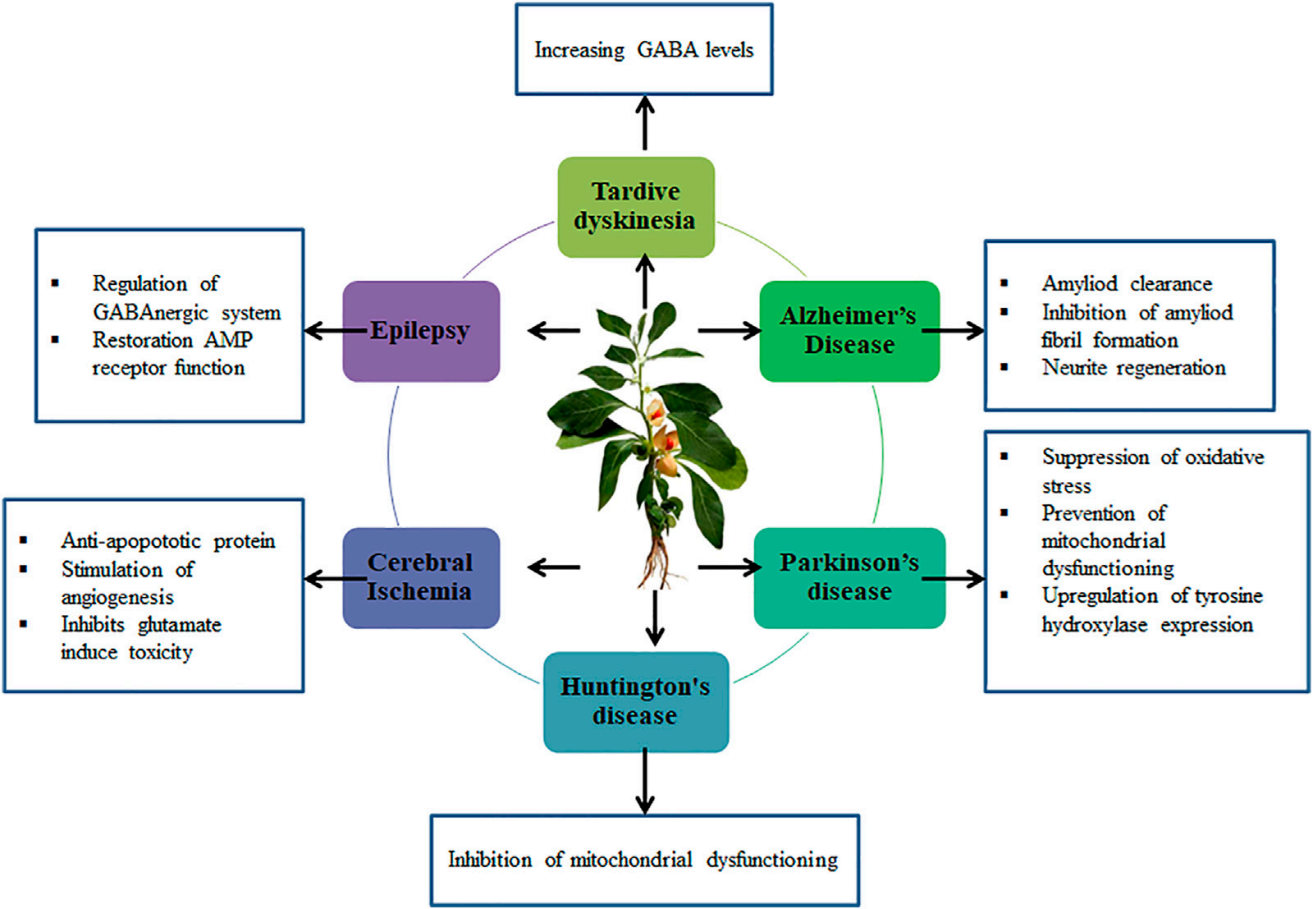
Overview of *Withania somnifera* on neurodegenerative diseases.

Epilepsy is a cognitive condition brought on by excessive neurotransmitter release. Depression, anxiety, and epilepsy are all prevalent conditions. Therefore, it is not surprising that a sizable number of people have both illnesses. Indeed, according to some experts, up to 55% of people with epilepsy will experience depression at some point in their lifetime (Jackson, 2005). Because so many people worldwide suffer from seizure disorders and there are so few effective treatments available, this condition is regarded as a major health problem. Around 15 million epileptic patients worldwide do not react to any of the treatments that are now available. Recurrent seizures are the hallmark of epilepsy. Glutamate and gamma amino butyric acid (GABA) are reported to play pivotal roles in the disordered balance of stimulatory and inhibitory neurotransmitters, which is the main cause of epilepsy (Barker-Haliski and White, 2015). *W*. *somnifera* shows anticonvulsant properties against pentylenetetrazol (PTZ) seizure threshold paradigm involved the GABAAergic modulation. *W*. *somnifera* (100 or 200 mg/kg) increases the PTZ threshold. Co administration of *W*. *somnifera* (50 mg/kg) with GABA (25 mg/kg) or diazepam (0.5 mg/kg) increased the seizure threshold (Kulkarni et al., 2008). The GABA receptor’s function is modulated and interfered by *W*. *somnifera* activating chloride channels, which increases the seizure threshold. The GABAergic neurotransmitter system is therefore most likely the fundamental mechanism by which the *W*. *somnifera* root extract raises the threshold of PTZ-induced seizure (Isoherranen et al., 2003). Co-administration of *W*. *somnifera* and Flax seed oil significantly reduces the convulsion phase in rats experiencing MES seizures (Tanna et al., 2012). Aqueous seed extracts of *W*. *somnifera* possess anti-convulsant by modulating the dopamine and serotonin levels in hippocampus of pilocarpine induced rat models (300 mg/kg) (Costa et al., 2012). Figures 4, 5 represents the effect of *W*. *somnifera* on different neurodegenerative diseases. *W*. *somnifera* produces bioactive natural chemicals that are rich in phenols, steroids, and flavonoid molecules and have good biocompatibility, bioavailability, and low toxicity. By regulating the endocrine, cardiac, central nervous system, and sexual behavior without causing any harm, Ashwagandha demonstrates a wide spectrum of therapeutic qualities. The root has been used most commonly for medicinal purposes and is a component of more than 200 Ayurvedic, Siddha, and Unani medicine formulations. Modern scientific research suggests that the aerial parts of the plant, such as its leaves, stem, fruit, and seeds, also possess several biologically active metabolites, despite the fact that the roots of the *W*. *somnifera* plant are the primary source of traditional Ayurvedic formulations. *W*. *somnifera* has been found to contain more than 40 withanolides, 12 alkaloids, and uncommon sitoindosides. The safety and clinical effectiveness of *W*. *somnifera* were documented in a total of 69 trials (30 clinical and 39 preclinical) (Kashyap et al., 2020). Numerous studies have reported that different parts of the plant have been linked to a variety of preclinical experiments, including cardioprotective (Khalil et al., 2015), anticancer (Alfaifi et al., 2016), antioxidant (Ahmed et al., 2018), antibacterial (Alam et al., 2012), antifungal (Bisht and Rawat, 2014), anti-inflammatory (Sahni and Srivastava, 1993), hepatoprotective (Sharma et al., 2021), anti-depressant (Bhattacharya et al., 2000), and hypoglycemic effects (Udayakumar et al., 2009). In a TPA-induced mice psoriatic-like paradigm, the concomitant administration of *W*. *somnifera* seed fatty acids decreased psoriatic lesions and skin inflammation. Extracts from seeds of *W*. *somnifera* has been shown to have potent anti-inflammatory activities by modifying NFκB activity and reducing the production of pro-inflammatory cytokines, such as IL-6 and TNF-, in cell-based experiments that were triggered by either TPA or LPS (Balkrishna et al., 2020).

## Future prospects

*W. somnifera* is one of the most widely used plant in Indian System of Medicine for several ailments and the claims for its use to improve a myriad of clinical conditions are overwhelmingly encouraging as a multi-purpose medicinal agent. However, at present given the paucity of randomized clinical trials (RCTs) there are insignificant number of clinically proven reports to justify its general medical use. We believe some of the questions are immediately warranted and are necessary before clinical recommendations on *W. somnifera* could be made confidently. Extracts of *W. somnifera* have been extensively studied in preclinical *in-vitro* and *in-vivo* models, demonstrating numerous molecular targets of *W. somnifera* and its effects in attenuating several dysfunctions and diseases in humans. But there are no comprehensive studies to decipher the effect of the active constitutes, rather there is a dichotomy in results in relation to several human disorders which could be answered by designing a well-regulated larger cohort of studies to analyze the effect of this potential drug candidate.
